# Scalp Melanoma Diagnosed by Fine Needle Aspiration Cytology in a Tertiary Health Center

**DOI:** 10.1155/2015/605906

**Published:** 2015-11-17

**Authors:** A. B. Zarami, N. A. Satumari, M. Ahmed

**Affiliations:** ^1^Department of Histopathology, University of Maiduguri Teaching Hospital, PMB 1414, Maiduguri, Borno State, Nigeria; ^2^Department of Medical Laboratory Science, University of Maiduguri, PMB 1069, Maiduguri, Borno State, Nigeria

## Abstract

Melanoma is one of the most aggressive malignant skin neoplasms worldwide with more than 20% of world melanoma seen in black Africa and Asia. Late presentation due to ignorance, poverty, and lack of adequate health facility in Nigeria is always the norms. We present this case report because of precision in diagnosis, using fine needle aspiration cytology (FNAC) to reemphasize that the technique is cheap, cost effective, and quick that can reduce the burden of incisional biopsy before definitive surgery and improve early detection of the disease especially in developing countries.

## 1. Introduction

Melanoma of skin accounts for 160,000 new cases annually; worldwide there were an estimated 41,000 deaths in 2002 alone [1], and it kills about 8000 Americans each year [2]. The mortality is more in men than in women [3], probably because the site distribution permits earlier diagnosis in females [3]. The neoplasm is generally common in women than in men and particularly common in White populations living in sunny climates [3]; this is thought to be due to ultraviolet radiation; in sub-Saharan Africa trauma is being implicated [4]. Most of the lesion is found in the head and neck area [5] and on the lower extremities, the latter location being particularly common in females. Other locations of cutaneous melanomas are the subungual region (“melanotic whitlow”) and the palms, soles, and nail bed [5].

The risk factors of the disease are being white, particularly those of fair complexion, red hair, and a tendency to burn or develop freckles after exposure to sunlight [5, 6]. The presence of a large number of melanocytic nevi represents a risk factor even if these nevi are not of the dysplastic type. The genetically determined disease xeroderma pigmentosum also predisposes to the development of melanoma. Cases of melanoma have been reported in association with type I Recklinghausen disease and in renal transplantation recipients [5].

Melanomas are divided into the following types: lentigo maligna, superficial spreading, acral lentiginous, mucosal, nodular, polypoid, desmoplastic, amelanotic, soft-tissue, melanoma with small nevus-like cells, melanoma with features of a spitz nevus, and uveal melanoma [7]. The superficial spreading type is the commonest and affects head and neck region; the commonest subtype of melanoma in Africa is the nodular variant that affects the feet [4]. The late presentation as always the custom in our environment and the accurate diagnosis using fine needle aspiration cytology in this index case prompted that case report.

## 2. Case Report

**Figure 1:**
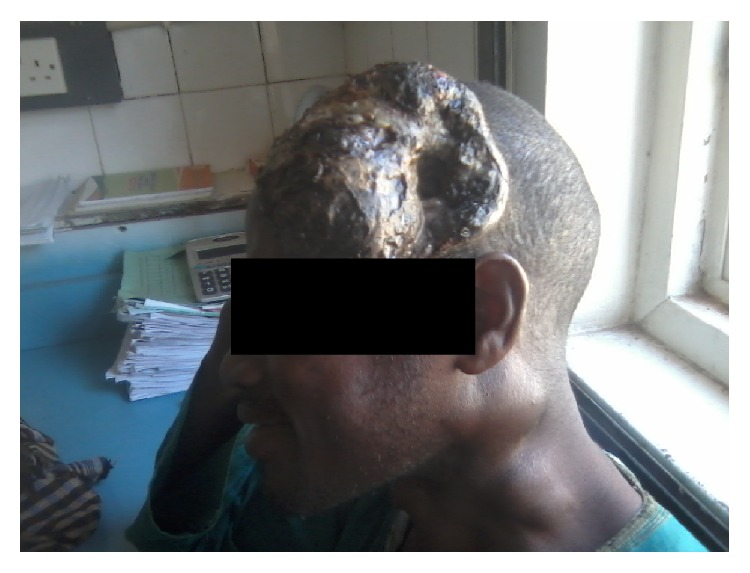


**Figure 2:**
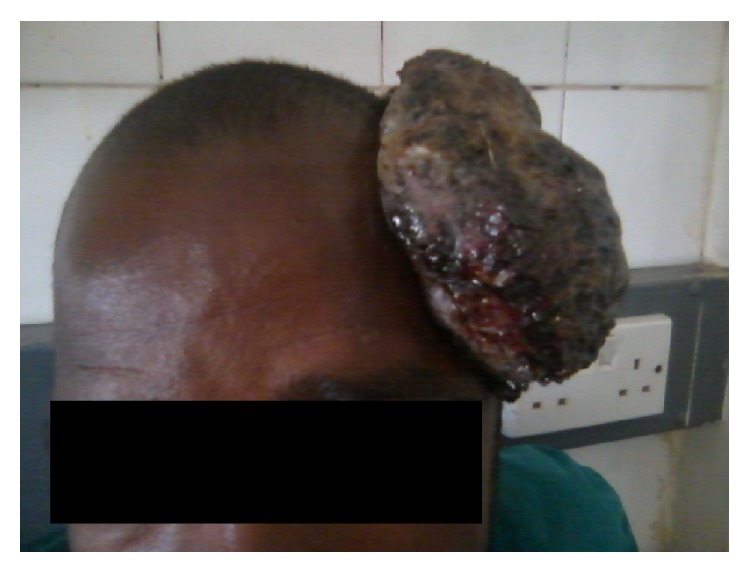


**Figure 3:**
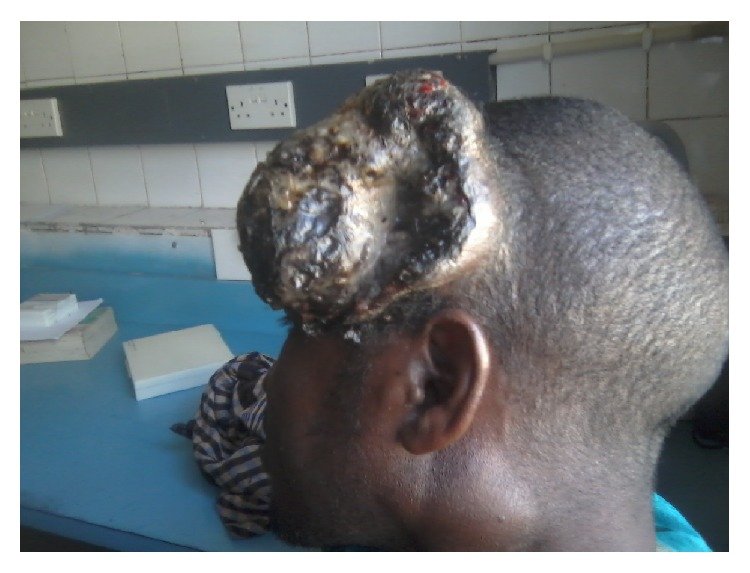


A 30-year-old man presented to our fine needle aspiration cytology (FNAC) clinic at University of Maiduguri Teaching Hospital (UMTH) from outpatient department. He presented with one-year history of scalp swelling and six-month history of cervical swelling; on examination we found scalp mass with variegated colour and ulcer measuring 14 × 12 cm, the floor is necrotic, and the edge is everted (Figures 1, 2, and 3). There are multiple cervical lymphadenopathies on the ipsilateral side, the largest measuring 4 cm in the widest diameter (Figures 1 and 3).

### 2.1. Procedure

The FNAC was done using Cameco (needle holder) with 20 mL syringe and 23-gauge needles. Aseptic procedure was observed and verbal consent was obtained from the patient. All ethical consideration was trail before carrying out the procedure and taking photograph. Aided by the Cameco, 0.5 mL of haemorrhagic grey aspirates was obtained. It was then immediately smeared on to a glass slide and then fixed in 95% alcohol for 15 min and then stained by haematoxylin and eosin (H and E). One of the slides was stained with Fontana Masson to demonstrate melanin (Figure 5). We are limited from performing immunohistochemistry, HMB 45, S-100, and Melan-A.

## 3. Cytology Report

**Figure 4:**
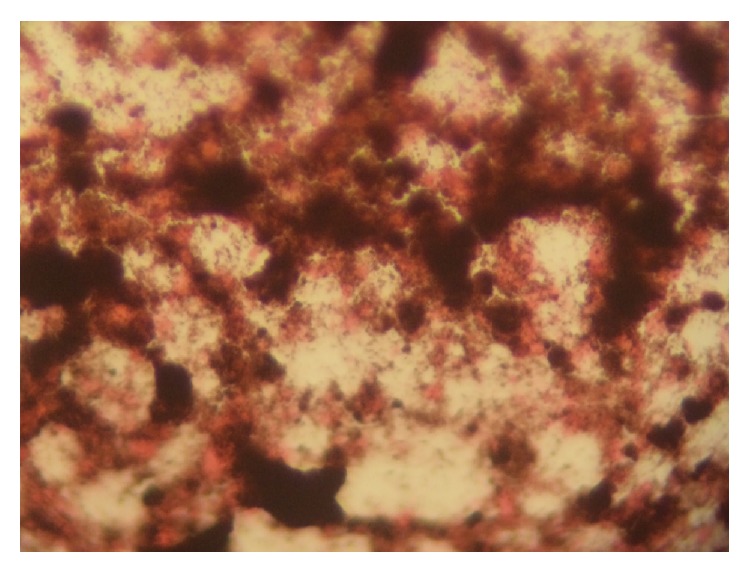
Photomicrograph of smear stained with H and E ×100.

**Figure 5:**
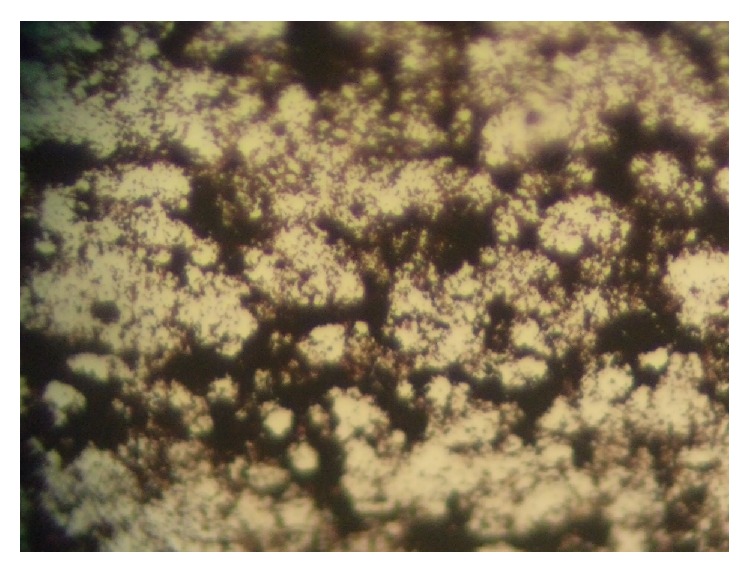
Photomicrograph of smear stained with Fontana Masson ×100.

Smears show abundant dark brown pigments along with few pleomorphic cells having round to oval nuclei with moderate cytoplasm. Quite a few cells that have prominent eosinophilic nucleolus each are present (Figure 4). A Fontana Masson stain was positive (Figure 5).

### 3.1. Diagnosis

Scalp mass/cervical lymph nodes were positive for malignancy (consistent with melanoma). 

## 4. Discussion

Melanoma is the commonest primary skin malignancy, the incidence of which is rising globally [4, 8, 9]. The incident has increased in the last twenty years at the rate of about 5% per year [8]. High rates of incidence are found in Australia/New Zealand, North America, and northern Europe [1]. Survival in developing countries is poorer (around 40%), in part due to late diagnosis and limited access to therapy but also because the tumours are generally acral located on the soles of the feet [1].

In Nigeria melanoma accounts for 34% of all skin malignancies, ranking second to squamous cell carcinoma of skin [9–11]. In a hospital base study in Maiduguri, 31% of all skin malignancies have been reported [4]. In another study in Cameroon, over nine-year period, melanomas represent 4.46% of all cancer and are the most frequent cancer [12]. The neoplasm has high cure rates when diagnosed early but poor survival when found at an advanced stage [13]. Most melanomas are visible on the skin, which allows for self-detection [13], though there is wide differential diagnosis. In a report based on a population-based survey, 53% of melanomas were detected by patients, 26% by physicians, 17% by family, and three percent by others [13]. Further, self-examination leads to decreased melanoma thickness in comparison to patients who do not perform self-examinations, with variable sensitivity data ranging from 25% to 93% but high specificity of 83% to 97% [3]. Despite the benefits of self-examination in early melanoma detection, this is often very difficult on the scalp [13]; late presentations especially in Nigeria are always the norm [4].

Fine needle aspiration cytology (FNAC) is cost-effective, quick, and accurate when used appropriately and has high precision in obtaining sample [13–15], even though its sensitivity and specificity depend on the types of the lesion; however, even rare cases like intraocular and oral melanoma have been diagnosed using FNAC [14, 15]. Nggada et al. reported sensitivity of 88.9% and specificity of 96.1% of FNAC of thyroid nodules over 10-year period with concordance rate of 94.2% [16]. In metastatic melanoma, a positive predictive value of 99% has been reported [17]. FNAC offers direct sampling and identification of the minority that are definite or probable malignancies and those that are follicular neoplasms, which require surgery with full histological assessment to exclude malignancy. Fine needle aspiration is most sensitive at detecting anaplastic (almost 100%) and papillary (around 90%) carcinomas of thyroid glands [13]. Although FNAC has greater accuracy in identifying tumours than alternative imaging or biochemical methods, it misses 5–10% of cancers. Even so, its incorporation into the diagnosis of thyroid nodules reduces the requirement for excision by at least 25% and doubles the yield of cancer in those that are excised [3, 13]. Core biopsy is more traumatic and has not been shown to increase accuracy of diagnosis [13].

Generally there is suboptimal utilization of FNAC in Nigeria despite its potential in diagnosis [18, 19]. Patient with a positive aspirate of palpable regional nodes like in the index case report can proceed directly to surgery, bypassing the need for an open biopsy [20]. Therefore, FNAC is an established tool for diagnosing many tumours. Its recognized complications that have been reported are rare, with an incidence of 0.003% to 0.07%, mostly from pancreatic tumours [21].

Molecular studies unveil multiple chromosomal aberrations in melanoma; the common changes include losses of 6q, 8q, 9p, and 10q and gain of 1q, 6p 7p, 8q, 11q, and 20q [22]. This pattern of changes depends on types of melanoma and the anatomical site of the lesion. The most common gene mutation in melanoma is* BRAF* and* NRAS* which accounted for 50% and 20%, respectively; both mutations increase activity of the MAPK pathway [23]. Thymine to adenine transversion is the basic abnormality that leads to substitution within the activation segment of serine/threonine kinase gene products. However,* BRAF* mutated pathway via p13k/AKT, which promotes survival and cell cycle entry in melanoma cells, is another mechanism.* PTEN* mutation, though uncommon, inhibits AKT-p13k pathways [23]. Cytogenetics or comparative genomic hybridization is highly essential for targeted therapy. To our knowledge there is no center that is doing that in developing countries of ours.

## 5. Conclusion

Intensification of public health education on melanoma especially in Africa can aid in early detection, even though, to date, no prospective evidence exists to clearly demonstrate the efficacy of prevention and early detection in decreasing melanoma mortality worldwide [24]. Nevertheless, many studies suggest that both self-assessment of risk factors and clinician examination can identify a proportion of patients at highest risk for melanoma who may benefit from behavior modification and routine screening [24]. Fine needle aspiration cytology has reduced initial biopsy before definitive surgery in most centers; its utility especially in developing countries of ours is highly encouraged despite our challenges so that the disease can be diagnosed early.
